# SCF E3 ligase PP2-B11 plays a positive role in response to salt stress in *Arabidopsis*

**DOI:** 10.1093/jxb/erv245

**Published:** 2015-06-02

**Authors:** Fengjuan Jia, Chunyan Wang, Jinguang Huang, Guodong Yang, Changai Wu, Chengchao Zheng

**Affiliations:** State Key Laboratory of Crop Biology, College of Life Sciences, Shandong Agricultural University, Taian, Shandong 271018, PR China

**Keywords:** AnnAt1, E3 AtPP2-B11, ligases, Na^+^ homeostasis, ROS.

## Abstract

Skp1–Cullin–F-box (SCF) E3 ligases are essential to the post-translational regulation of many important factors involved in cellular signal transduction. In this study, we identified an F-box protein from *Arabidopsis thaliana*, AtPP2-B11, which was remarkably induced with increased duration of salt treatment in terms of both transcript and protein levels. Transgenic *Arabidopsis* plants overexpressing *AtPP2-B11* exhibited obvious tolerance to high salinity, whereas the RNA interference line was more sensitive to salt stress than wild-type plants. Isobaric tag for relative and absolute quantification analysis revealed that 4311 differentially expressed proteins were regulated by AtPP2-B11 under salt stress. AtPP2-B11 could upregulate the expression of annexin1 (AnnAt1) and function as a molecular link between salt stress and reactive oxygen species accumulation in *Arabidopsis*. Moreover, *AtPP2-B11* influenced the expression of Na^+^ homeostasis genes under salt stress, and the *AtPP2-B11* overexpressing lines exhibited lower Na^+^ accumulation. These results suggest that *AtPP2-B11* functions as a positive regulator in response to salt stress in *Arabidopsis*.

## Highlight

AtPP2-B11, an F-box protein, enhances the salt stress tolerance by regulating *AnnAt1* expression, repressing reactive oxygen species production, and disrupting Na^+^ homeostasis in *Arabidopsis*.

## Introduction

Soil salinity has an important effect on plant growth, plant development, and crop productivity (Zhu, 2001). More than one-fifth of the world’s arable land is currently under the threat of salt stress, and plants must cope with salt stress during their life cycle (Li *et al.*, 2011). Ion imbalance and osmotic stress can be caused by high concentrations of salts in plants. The high accumulation of Na^+^ can disrupt K^+^ nutrition and hinder the functions of many enzymes in the cytoplasm, resulting in plant growth inhibition (Xiong *et al.*, 2002). To survive environmental salt stress, plants have developed various mechanisms to perceive external signals and maintain intracellular ionic homeostasis of Na^+^/ K^+^ (Zhu, 2001, 2002, 2003). A previous study reported that the salt stress-induced salt overly sensitive (SOS) pathway is an important regulatory system for plant Na^+^/K^+^ homeostasis in *Arabidopsis thaliana* (Zhu, 2003). The SOS pathway can be activated by salt stress to maintain high K^+^ and low Na^+^ homeostasis for salt tolerance, and three components have been defined, SOS1, SOS2, and SOS3 (Zhu *et al.*, 1998; Zhu, 2003). SOS3 is a calcium sensor that contains four EF-hand domains and an *N*-myristoylation signal peptide (Liu and Zhu, 1998; Ishitani *et al.*, 2000; Sanchez-Barrena *et al.*, 2005). SOS3 decodes a calcium signal that can perceive the increase in intracellular calcium stimulated by salt stress and then recruits and activates SOS2, a protein kinase (Halfter *et al.*, 2000; Liu *et al.*, 2000; Guo *et al.*, 2001, 2004). Subsequently, the SOS3–SOS2 complex localizes to the plasma membrane and activates SOS1, a plasma membrane Na^+^/H^+^ antiporter, to transport Na^+^ out of the cell (Shi *et al.*, 2000, 2002). Ion channels, including the Na^+^/H^+^ antiporter NHX, voltage-dependent cation channels HKT and KUP, proton pumps H^+^-ATPase and H^+^-pyrophosphatase, and Ca^2+^/H^+^ antiporter VCX1, also play important roles in Na^+^ compartmentation (Gaxiola *et al.*, 2001; Senn *et al.*, 2001; Berthomieu *et al.*, 2003; An *et al.*, 2007; Fuglsang *et al.*, 2007; Yang *et al.*, 2010; Barragan *et al.*, 2012). The phytohormone abscisic acid (ABA) exerts a significant function for coping with salt stress. The induced expression of many genes that appear to play multifaceted roles in salt response and tolerance is caused by the increased ABA levels when stressed with high salinity (Finkelstein *et al.*, 2002). The products of ABA-induced genes include late embryogenesis abundant (LEA) proteins; regulatory proteins such as transcription factors, protein kinases, and phosphatases; various transporters; and enzymes involved in osmoprotectant synthesis, phospholipid signalling, fatty acid metabolism, and cellular metabolism (Seki *et al.*, 2002; Nemhauser *et al.*, 2006; Cutler *et al.*, 2010; Fujita *et al.*, 2011).

Besides the primary effects caused by high salinity, secondary stresses such as oxidative damage often occur (Zhu, 2001). Increased reactive oxygen species (ROS) formation is observed in plants in response to both osmotic and ionic stresses associated with soil salinity, which secondarily results in oxidative stress and cell damage (Flowers, 2004; Miller *et al.*, 2010; Xie *et al.*, 2011). ROS, including hydrogen peroxide (H_2_O_2_), superoxide anion, and hydroxyl radical (Kovtun *et al.*, 2000), can damage most cellular macromolecules directly, and cause irreversible damage and hypersensitive response-like cell death (Apel and Hirt, 2004). On the other hand, ROS have also been proposed to act as a signalling mediator of plant salinity tolerance (Kwak *et al.*, 2003). Moreover, high extracellular NaCl causes a Ca^2+^ influx to elevate cytosolic free Ca^2+^ ([Ca^2+^]_cyt_) as a secondary messenger for adaptive signalling in plant cells (Kiegle *et al.*, 2000; Shi *et al.*, 2000; Tracy *et al.*, 2008). Plant annexins are typical stress response genes, which can form Ca^2+^-permeable conductance in an oxidized membrane to simulate ROS signalling. Annexin is a multigene, multifunctional family of Ca^2+^-dependent membrane-binding proteins found in both animal and plant cells (Mortimer *et al.*, 2008). In the *Arabidopsis* genome, eight annexin proteins have been identified (Cantero *et al.*, 2006). The expression levels of all eight annexin genes in *Arabidopsis* are differentially regulated by drought, salt, and other abiotic stresses (Cantero *et al.*, 2006). *AnnAt1* is currently the best characterized of the eight annexins, and its expression is the most abundant in *Arabidopsis* (Konopka-Postupolska *et al.*, 2009). T-DNA insertion mutants of *AnnAt1* play an important role during germination under salt and osmotic stresses, and the impaired ability of the *AnnAt1* mutants to germinate under stress conditions is rescued by complementation with wild-type *AnnAt1* (Lee *et al.*, 2004). Further studies have demonstrated that *AnnAt1* responds to high extracellular NaCl by mediating ROS-activated Ca^2+^ influx across the plasma membrane of root epidermal protoplasts, providing a molecular link between ROS and cytosolic Ca^2+^ in plants (Laohavisit *et al.*, 2012, 2013). Given the low levels of peroxidase activity, expression of *AnnAt1* in the ΔoxyR mutant of *Escherichia coli* sensitive to H_2_O_2_ greatly increases the organism’s survivability in the presence of H_2_O_2_ (Gidrol *et al.*, 1996). However, the regulators of *Arabidopsis AnnAt1* under salt stress remain unknown.

In eukaryotes, protein degradation by the ubiquitin (Ub)-mediated regulation pathway is one of the key mechanisms for protein metabolism (Sadanandom *et al.*, 2012). Ub is a small protein that contains 76 aa and is highly conserved in different species. Three enzymes, E1 (Ub-activating enzyme), E2 (Ub-conjugating enzyme), and E3 (Ub-protein ligases), can catalyse the Ub molecule attached to its target protein through sequential actions (Sadanandom *et al.*, 2012). Among these three enzymes, E3s are the most diverse proteins in the Ub-mediated protein degradation pathway to confer substrate selectivity for an extensive range of substrates (Moon *et al.*, 2004; Sadanandom *et al.*, 2012). Approximately 1406 E3 genes have been identified in *Arabidopsis*, and these E3s can be subdivided into two basic groups based on the occurrence of either a ‘Homology to E6-AP C-Terminus’ (HECT) or ‘Really Interesting New Gene’ (RING)/U-box domain (Vierstra, 2009; Sadanandom *et al.*, 2012). Skp1–Cullin–F-box (SCF), belonging to the RING-containing proteins, functions as a multi-subunit E3 complex. The SCF complex is composed of four subunits, SKP1 (ASK in plants for *Arabidopsis* SKP1), CDC53 (or Cullin), F-box protein, and the RING finger protein RBX1 (for Ring-Box 1) (Moon *et al.*, 2004). Among these four subunits, the F-box protein plays an important role in binding a diverse array of substrates and is one of the largest gene families in plants. In *Arabidopsis*, 694 potential F-box proteins have been identified, whereas 687 and 359 potential F-box proteins have been identified in rice and maize, respectively (Jain *et al.*, 2007; Jia *et al.*, 2013). F-box proteins usually contain a conserved F-box domain (40–50 aa) at their N terminus, which interacts with Skp1. At their C terminus, they generally contain one or several highly variable protein–protein interaction domains, such as tubby, FBD, WD40, LRR, FBA, kelch repeats, DUF, and JmjC (Gagne *et al.*, 2002; Kuroda *et al.*, 2002). The F-box protein is a key regulatory protein in many cellular processes, including hormone response, photomorphogenesis, floral development, senescence, and various signal transduction pathways (Moon *et al.*, 2004; Sadanandom *et al.*, 2012). However, information on F-box genes regulating abiotic stress is limited.

Our previous study demonstrated that AtPP2-B11, an SCF E3 ligase, plays a role in the response to drought stress as a negative regulator in *Arabidopsis*. An AtPP2-B11-interacting protein, AtLEA14, was identified, and overexpressing *AtPP2-B11* led to a decrease in the expression levels of AtLEA14 in both the transcript and protein levels when under drought stress conditions (Li *et al.*, 2013). However, evidence of an *AtPP2-B11*-mediated salt stress response is unknown. In the present study, cytoplasm-localized AtPP2-B11, which was induced by salt stress, enhanced salt stress tolerance during seed germination and in mature plants, whereas the RNA interference (RNAi) line exhibited obvious sensitivity to salt stress. Isobaric tag for relative and absolute quantification (iTRAQ) analysis provided evidence of the involvement of annexins in salt stress tolerance in *AtPP2-B11* overexpressing lines. Our results suggest that *AtPP2-B11* plays an important role in the response to salt stress by regulating *AnnAt1* expression, repressing ROS production, and disrupting Na^+^ homeostasis in *Arabidopsis*.

## Materials and methods

### Plant materials and growth conditions

*Arabidopsis thaliana* ecotype Columbia (Col-0) was used in this study. Seeds were surface-sterilized by soaking in 70% ethanol for 5min and 10% NaClO for 10min, and then washed seven times with sterilized water. The sterile seeds were plated on ½

Murashige and Skoog (MS) medium containing 1% (w/v) sucrose. The plates were then kept in the dark at 4 ° for stratification for 3 d before being placed in a growth room for germination. Seedlings were transferred to soil and grown to maturity at 22 °C with a 16h light/8h dark cycle.

### Construction and generation of transgenic plants

To construct *35S::AtPP2-B11*, the *AtPP2-B11* coding sequence was amplified using Col-0 cDNA by PCR with gene-specific primers (Supplementary Table S5, available at *JXB* online). The resulting PCR product was cloned into the *Eco*RI and *Bam*HI sites of binary vector pBI121 under the control of a cauliflower mosaic virus 35S promoter. To generate an *AtPP2-B11* RNAi construct, *Arabidopsis* pFGC5941 vector for dsRNA production was used. A 432bp fragment of *AtPP2-B11* cDNA was amplified by PCR using gene-specific primers (Supplementary Table S5). The fragment was initially cloned between the *Asc*I and *Swa*I sites before an inverted repeat of the same fragment was inserted between the *Bam*HI and *Xba*I sites of pFGC5941. The transformation of *Arabidopsis* plants was performed by floral dip using *Agrobacterium tumefaciens* strain GV3101. Homozygous T3 lines were used for phenotypic analysis.

### Seed germination and salt phenotype analysis

A seed germination assay was performed with 100 seeds and repeated three times. Seeds of wild-type, *35S::AtPP2-B11* lines and the RNAi line were grown on ½ MS medium supplemented with 150 or 200mM NaCl at 22 °C with a 16h light/8h dark cycle. Germination was defined as obvious emergence of the radicle through the seed coat. Wild-type, *35S::AtPP2-B11* lines, and RNAi plants were grown for 4 weeks under normal growth conditions and then subjected to salt stress by watering with 200mM NaCl for 20 d. Three days after rewatering, surviving plants were counted.

### RNA extraction and real-time reverse transcription (RT)-PCR analysis

To assay the relative expressions of genes, real-time RT-PCR analysis was performed with the RNA samples isolated from 2-week-old seedlings after treatment with salt stress. Total RNA was extracted with Trizol reagent (Invitrogen, USA). All samples were made up to 10 µl in volume. A SYBR Green Real-time PCR Master Mix (Takara, Japan) and a Chromo 4 Real-time PCR detector (Bio-Rad, USA) were used for real-time RT-PCR. The data are presented after normalizing to the reference gene, glyceraldehyde 3-phosphate dehydrogenase (*GAPDH*), and three biological replicates under similar conditions were performed for each experiment. The specific primers for these genes are listed in Supplementary Table S5.

### Histochemical staining

To detect the contents of H_2_O_2_ and O^2–^ in *Arabidopsis*, plants were fixed with 3′,3′-diaminobenzidine (DAB) or nitro blue tetrazolium (NBT), respectively, as described by Orozco-Cardenas and Ryan (1999) and Kawai-Yamada *et al.* (2004). The 2-week-old seedlings of OE10, OE11, R-5, and wild-type plants were treated with 200mM NaCl for 6h and then incubated with DAB or NBT for 8h at 25 °C in the dark (covered with aluminium foil). Stained samples were cleared by boiling using stain fixative (acetic acid:glycerol:ethanol, 1:1:3, v/v/v) before photographs were taken.

### Western blot assays

Antibody specific for AtPP2-B11, AnnAt1, and AtLEA14 were produced by CWBIO (China), using the specific oligopeptides of each protein to immunize rabbit. Plant total proteins were extracted using a Plant Protein Extraction Kit (CWBIO). Next, 30 µg of total protein per lane was separated by 15% SDS-PAGE. After electrophoresis, the proteins were electrotransferred to polyvinylidene difluoride membrane. After blocking for 3h in TBST (50mM Tris/HCl, pH 7.5, 150mM NaCl, 0.05% Tween 20) with 5% non-fat dried milk at room temperature, membranes were incubated with antibody at a 1:1000 dilution for 2h. After three washes with TBST, the membranes were incubated with IgG (CWBIO) for 1h. After three washes with TBST, the membranes were visualized using an enhanced Lumi-Light Western Blotting Substrate kit (Thermo Scientific, USA) following the manufacturer’s instructions. The protein levels were quantified using ImageJ software.

### Expression and purification of AtLEA14

AtLEA14 was cloned into pET30a and the recombinant plasmid was transformed into *E. coli* BL21 competent cells. When OD_600_ of 0.4–0.6 was reached, isopropyl-β-d-thiogalactoside was added to the LB culture to a final concentration of 1mM. After incubation at 16 °C for 8h, the bacterial cells were harvested, and recombinant AtLEA14 was purified using a 6×His-tagged Protein Purification kit (CWBIO).

### Chlorophyll content measurement

Plant leaves were collected, their fresh weight determined and washed in distilled water. Chlorophyll was extracted in 80% (v/v) acetone at 25 °C in the dark for 24h, and the concentration of chlorophyll *a*/*b* was determined according to Komatsu *et al.* (2010).

### Glutathione measurement

Spectrophotometric plate reader assay was performed to determine the oxidized and reduced forms of glutathione using Corning 96-well UV-transparent plates with an A061-1 kit (Nanjing Jiancheng, China). Standards and sample extracts were assayed in triplicate.

### Na^+^ distribution visualization and Na^+^ content measurement

Na^+^ distribution was visualized with CoroNa Green-AM (Invitrogen). Two-week-old seedlings were treated with 200mM NaCl for 6h and then incubated with 2.5 μM CoroNa Green-AM for 3h in the dark. *Z*-stacks of leaf fluorescent were observed and recorded with a Zeiss LSM 510 confocal system. For measurement of Na^+^ content in seedlings, 2-week-old plants were harvested, dried for 48h at 80 °C and then ground to a powder. The same mass of tissue powder was digested in concentrated (69%, v/v) HNO_3_ for 24h at room temperature for elemental extraction. Na^+^ concentration was determined by atomic absorption spectrophotometry.

### *AnnAt1* promoter cloning and luminescence imaging

The promoter sequence of *AnnAt1* was amplified by PCR using the primers 5′-AAGCTTAAACTGAACTTCTCCATCAATTTC-3′ and 5′-GGATCCCTTCTACTTTTAGTGTTTTGTGTATG-3′. The resulting fragment was cloned into the *Bam*HI and *Hin*dⅢ sites of the vector pGWB535 to generate a fusion construct with luciferase (LUC). This vector was introduced into *A. tumefaciens* strain GV3101. For infiltration of *Nicotiana benthamiana*, the P19 protein of tomato bushy stunt virus was used to suppress gene silencing (Voinnet *et al.*, 2003). Co-infiltration of *A. tumefaciens* strains containing the *pAnnAT1::LUC* (*pA::LUC*) construct and the P19 silencing plasmid with or without the *35S::AtPP2-B11* construct was infiltrated into leaves of 4-week-old *N. benthamiana* plants as described previously (Walter *et al.*, 2004). For luminescence imaging, the plants were sprayed with 1mM d-luciferin (Wako, Japan) containing 0.01% Triton X-100. After 5min in the dark, the luminescence was observed using an LAS3000 imager (Fujifilm, Japan) and an LAS4000 imager (GE Healthcare, Japan).

## Results

### Expression of *AtPP2-B11* is upregulated by salt stress

**Fig. 1.**
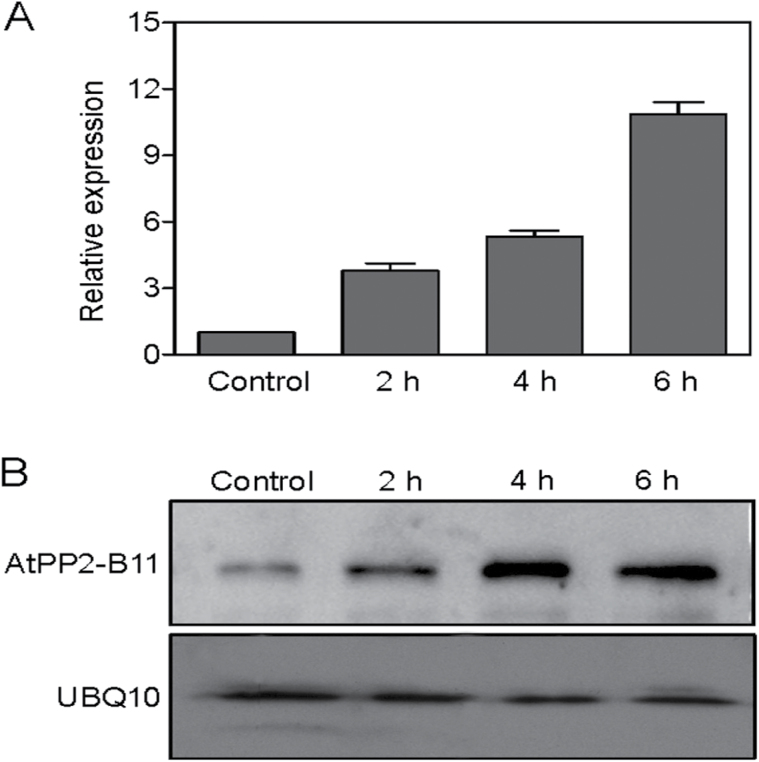
Expression of *AtPP2-B11* is induced by salt stress. (A). Induction investigation of *AtPP2-B11* by salt stress at the transcriptional level. Total RNA from 2-week-old wild-type plants was obtained and analysed by real-time RT-PCR. The mean values of real-time RT-PCR from three biological replicates were normalized to the levels of an internal control, *GAPDH*. Error bar indicates SD (*n*=3). (B). Induction investigation of AtPP2-B11 by salt stress at the protein level. Total proteins from 2-week-old wild-type plants were obtained and analysed by Western blotting. Experiments were repeated three times with similar results. UBQ10 was used as a loading control.

To investigate the expression pattern of *AtPP2-B11* under salt stress conditions, the transcript levels of *AtPP2-B11* were detected by real-time RT-PCR. As shown in Fig. 1A, the expression of *AtPP2-B11* in *Arabidopsis* seedlings was induced by treatment with 200mM NaCl. Consistent with this finding, a drastic increase in AtPP2-B11 protein was also observed with increased duration of salt treatment (Fig. 1B). The salt-induced expression of *AtPP2-B11* at both the transcriptional and translational levels led us to hypothesize that *AtPP2-B11* might be involved in the response to salt stress in plants.

### *AtPP2-B11* overexpressing *Arabidopsis* enhances salt tolerance at the seedling stage

**Fig. 2.**
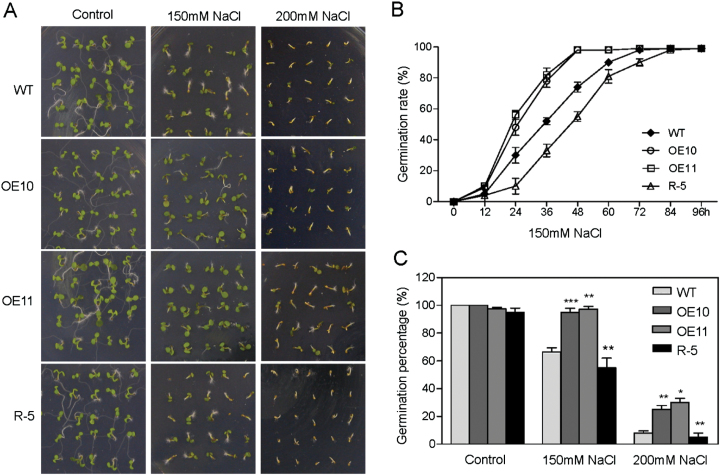
Phenotypes of *AtPP2-B11* transgenic (OE10, OE11, and R-5) and wild-type (WT) plants at the germination stage under salt stress conditions. (A) Salt response of *AtPP2-B11* transgenic and wild-type plants at the germination stage. (B) Seed germination rates of *AtPP2-B11* transgenic and wild-type plants grown on ½ MS medium containing 150mM NaCl. (C) Seed germination percentages of *AtPP2-B11* transgenic and wild-type plants grown on ½ MS medium containing different NaCl concentrations 2 d after germination. Germination rates in terms of an obvious emergence of the radicle through the seed coat were determined after the end of stratification. Data in (B) and (C) show the mean±SD of at least three replicates. For each replicate, 100 seeds per genotype were counted. (This figure is available in colour at *JXB* online.)

To test the biological function of *AtPP2-B11* in *Arabidopsis*, we regenerated both *AtPP2-B11* overexpression transgenic lines and RNAi lines for further study. Homozygous transformant seedlings were screened, and AtPP2-B11 mRNA and proteins levels of these transgenic *Arabidopsis* lines were confirmed by real-time RT-PCR and Western blotting. Two overexpressing (OE) lines (OE10 and OE11) and one RNAi line (R-5) were selected for further experiments (Supplementary Fig. S1, available at *JXB* online). Sterilized seeds of OE10, OE11, R-5, and wild-type plants were plated on ½ MS growth medium or ½ MS medium containing 150 or 200mM NaCl, respectively. The results showed that OE10 and OE11 exhibited more salt stress tolerance than wild-type and R-5 plants at the seedling stage (Fig. 2A). The seed germination rates of transgenic *Arabidopsis* (OE10, OE11, and R-5) exhibited no significant differences from wild-type plants when grown under normal conditions (Fig. 2A). However, under salt stress conditions with 150mM NaCl, the two OE lines (OE10 and OE11) showed ~30% higher seed germination than wild-type plants 2 d after germination, while the R-5 line exhibited ~10% lower germination rate than wild-type plants (Fig. 2B). When the NaCl concentration was increased to 200mM, the germination rates of these four genotype seeds decreased drastically. However, the seed germination percentages of *AtPP2-B11* overexpressing lines were much higher than those of wild type and the R-5 line (Fig. 2C). Although there was a delay in germination of R-5 and wild-type compared with AtPP2-B11 overexpressing lines under salt stress conditions, the germination rates overall were not affected in OE10, OE11, R-5 and wild-type plants.

**Fig. 3.**
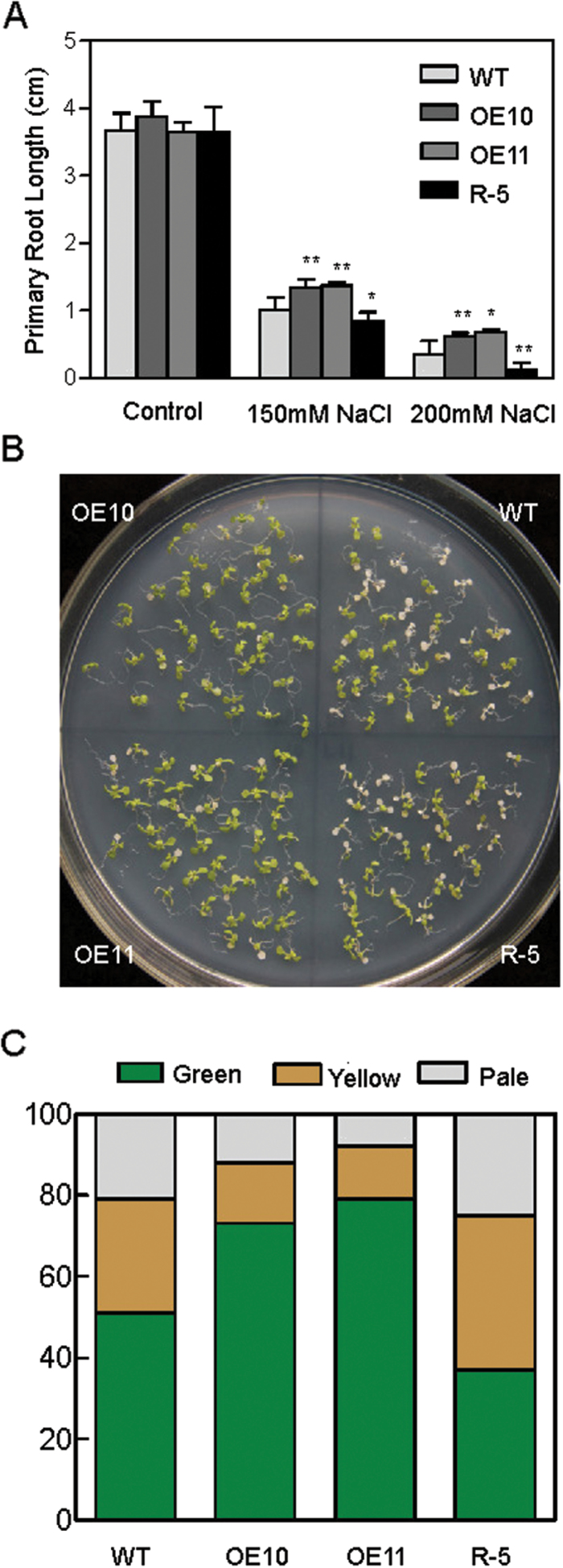
Responses of *AtPP2-B11* transgenic and wild-type (WT) plants to salt stress in the seedling stage. (A) Primary root length of 2-week-old plants on ½ MS medium containing different NaCl concentrations. Data show the mean±SD of at least three replicates. (B) Photograph of the *AtPP2-B11* transgenic and wild-type plants taken 7 d after being transferred to ½ MS medium containing 200mM NaCl. (C) Percentages of green, yellow, and pale seedlings of different genotypes exposed to 200mM NaCl. (This figure is available in colour at *JXB* online.)

In order to explore the effect of salt stress on the growth of *AtPP2-B11* transgenic lines at the seedling stage, root growth inhibition was measured on vertical growth plates with different concentrations of NaCl. After treatment for 2 weeks with NaCl, similar phenotypes were observed where the root length of *AtPP2-B11* overexpressing plants was significantly longer than that of wild-type plants and the R-5 line, while no differences were observed when plants were grown on ½ MS agar medium (Fig. 3A). To confirm the increased tolerance of *AtPP2-B11* to salt, the post-germination growth of transgenic plants was also investigated. The transgenic lines OE10, OE11, and R-5 and wild-type plants were allowed to grow on ½ MS medium for 7 d and then transferred to ½ MS medium containing 200mM NaCl for a further 7 d of growth. The percentages of green seedlings were 74 and 78% for OE10 and OE11, while wild-type and R-5 lines were only 51 and 37%, respectively (Figs. 3B, C). These results indicated that the *AtPP2-B11* overexpressing lines were more tolerant to salt stress than wild-type plants both at the germination and post-germination stages, whereas the RNAi line was more sensitive to salt stress.

### *AtPP2-B11* overexpressing *Arabidopsis* enhances salt tolerance at the adult stage

**Fig. 4.**
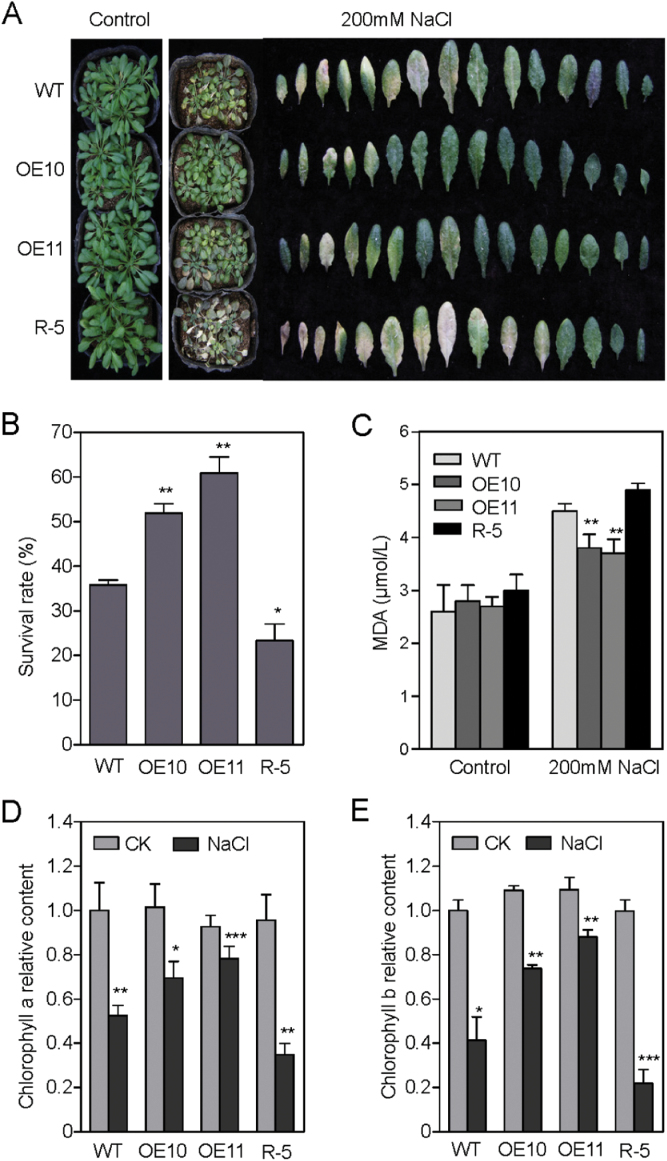
Responses of *AtPP2-B11* transgenic and wild-type (WT) plants to salt stress at the adult stage. (A) Photographs of *AtPP2-B11* transgenic and wild-type plants after watering with 200mM NaCl. The transgenic and wild-type plants were transferred to soil grown for 4 weeks under normal growth conditions and then watered with 200mM NaCl for 20 d. (B) Survival rates of *AtPP2-B11* transgenic and wild-type plants. Survival rates were determined after 3 d of rewatering. Fifty plants were tested in each experiment. (C) MDA contents of *AtPP2-B11* transgenic and wild-type plants. Four-week-old *AtPP2-B11* transgenic and wild-type plants were treated with 200mM NaCl for 6h, and water replaced the NaCl as a control. (D, E). Chlorophyll *a* (D) and *b* (E) levels of *AtPP2-B11* transgenic and wild-type plants before and after watering with 200mM NaCl for 10 d at the adult stage. Data showed the mean±SD of at least three replicates. (This figure is available in colour at *JXB* online.)

To investigate further whether *AtPP2-B11* functions at the adult stage, adult plants of the OE10, OE11, and R-5 lines and wild-type plants were tested for their response to high salinity. The seeds of these four genotypes were plated on ½ MS for 7 d and then transferred to soil growth for 4 weeks. Healthy and uniform transgenic and wild-type plants were selected for further treatment with or without 200mM NaCl for 20 d. Under normal growth conditions, the transgenic lines and wild-type plants showed no significant morphological or developmental abnormalities. However, when subjected to salt stress, the OE10 and OE11 lines had much greener and truer leaves, whereas a higher percentage of pale seedlings was observed in R-5 plants (Fig. 4A). Three days after rewatering, the survival ratios of the OE10 and OE11 lines were 51 and 60%, while the survival ratio of R-5 and wild-type plants were only 23 and 35%, respectively (Fig. 4B). The content of malondialdehyde (MDA) was also measured in *AtPP2-B11* transgenic plants and wild-type plants, which could represent the oxidative level of membrane lipid. Although the accumulation of MDA in all four genotypes was significantly elevated under salt stress, the OE10 and OE11 lines had lower MDA levels than the wild-type and R-5 plants (Fig. 4C). In addition, the levels of chlorophyll of transgenic *Arabidopsis* and wild-type plants were similar under normal growth conditions. However, under salt growth conditions, the OE10 and OE11 lines had higher levels of chlorophyll *a* and chlorophyll *b* than the wild-type and R-5 plants (Fig. 4D, E). These results indicated that *AtPP2-B11* acts as a positive regulator in response to salt at both the seedling and adult stages.

### Quantitative identification of AtPP2-B11-responsive proteins under salt stress conditions

**Fig. 5.**
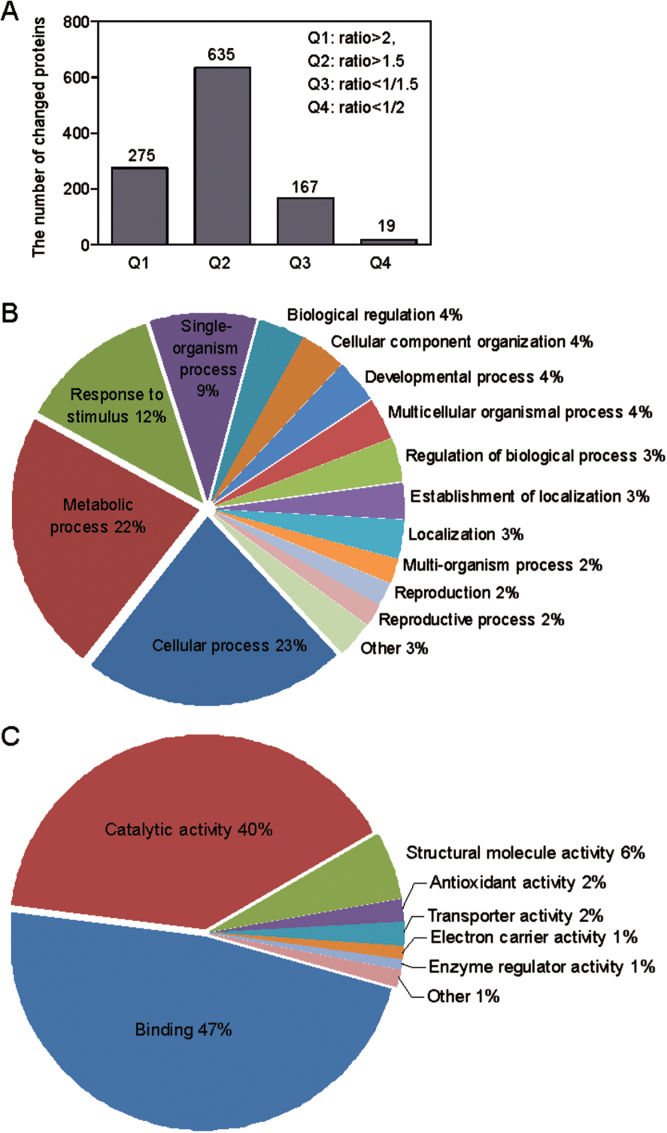
GO analysis of proteins upregulated by twofold in the OE11 line compared with those in wild-type plants under salt stress conditions. (A). Numbers of differentially expressed proteins of the OE11 line compared with those of wild-type plants. The ratios were >2, >1.5, <1/1.5 and <1/2. (B). ‘Biological process’ analysis of the twofold upregulated proteins. (C). ‘Molecular function’ analysis of the twofold upregulated proteins. (This figure is available in colour at *JXB* online.)

To elucidate the molecular mechanism of *AtPP2-B11* in response to salt stress in plants, we compared the protein profile changes between OE11 and wild-type plants using the iTRAQ technique. Since the root length and post-germination growth of 2-week-old *AtPP2-B11* overexpressing plants all exhibited more tolerance to salt stress than wild-type plants, total protein of 2-week-old OE11 and wild-type plants under salt stress for 6h was extracted using a urea/CHAPS extraction buffer, digested in solution, labelled with iTRAQ reagents, and quantitatively identified by LTQ-Orbitrap XL hybrid MS scans (Supplementary Fig. S2, available at *JXB* online). A total of 5881 proteins were identified in the two experiments and 4311 proteins were quantitated, covering a wide range of metabolic and signalling pathways (Supplementary Table S1, available at *JXB* online). Compared with the wild-type plants, various proteins in the OE11 plants exhibited significant changes. At a threshold of 2-fold change, 275 and 19 proteins were up- and downregulated, while with a threshold of 1.5-fold change, a total of 635 and 167 proteins were up- and downregulated, respectively (Fig. 5A). Interestingly, the number of upregulated proteins was much higher than that of downregulated ones, indicating that *AtPP2-B11* probably play positive roles in regulating other proteins under salt stress conditions.

Gene Ontology (GO) analysis of the 2-fold upregulated proteins in the iTRAQ system revealed that the majority of the most abundant proteins in *Arabidopsis* were related to basic metabolism, as demonstrated in previous studies. For example, under the term ‘Biological processes’, the highest proportions of upregulated proteins were ‘Cellular process’ (23%) and ‘Metabolic process’ (22%) (Fig. 5B). After these was ‘Response to stimulus’ (12%) (biotic, abiotic, and endogenous stimuli, etc.), indicating that *AtPP2-B11* may play an important role in stress resistance (Fig. 5B). Analysis of the GO term ‘Molecular function’ showed that, except for the high proportion of ‘Binding’ (47%), ‘Catalytic activity’ (40%), and ‘Structural molecule activity’ (6%), ‘Antioxidant activity’ (2%) and ‘Transporter activity’ (2%) had the highest proportion of upregulated proteins under salt stress (Fig. 5C). Proteins that were upregulated upon high salinity were enriched in the GO categories ‘Response to stimulus’ (88 proteins) and ‘Antioxidant activity’ (seven proteins), demonstrating that *AtPP2-B11* may be involved in salt tolerance by regulating the stress-responsive proteins and redox status in *Arabidopsis*.

In order to investigate further the proteins involved in salt stress tolerance, the response of proteins to salt stress and oxidative stress with a threshold of 1.5-fold change were determined (Supplementary Tables S2 and S3, available at *JXB* online). The proteins participating in salt and oxidative stresses had internal contact, and some proteins, including P5CS1, APX3, and CAT2, were upregulated in the OE11 line with these two stresses at the same time. The upregulated basic metabolic processes, such as photosynthesis, the citrate cycle, and oxidative phosphorylation, would also contribute to the high tolerance of *AtPP2-B11* overexpressing plants to environment stresses (Supplementary Table S2). Salt stress usually triggers the elevation of cytosolic Ca^2+^ level in plant cells, and in the OE11 line, the response of many proteins to cadmium ions was upregulated, indicating that the secondary messenger Ca^2+^ participated in the *AtPP2-B11*-regulated increase in salt stress tolerance (Supplementary Table S2). Many proteins involved in oxidative stress were also upregulated in the OE11 line under salt stress conditions, such as proteins response to oxidative stress, the response to H_2_O_2_, proteins involving in oxygen and ROS metabolic processes, the electron transport chain and oxidative phosphorylation (Supplementary Table S3).

### AnnAt1 is upregulated by AtPP2-B11

**Fig. 6.**
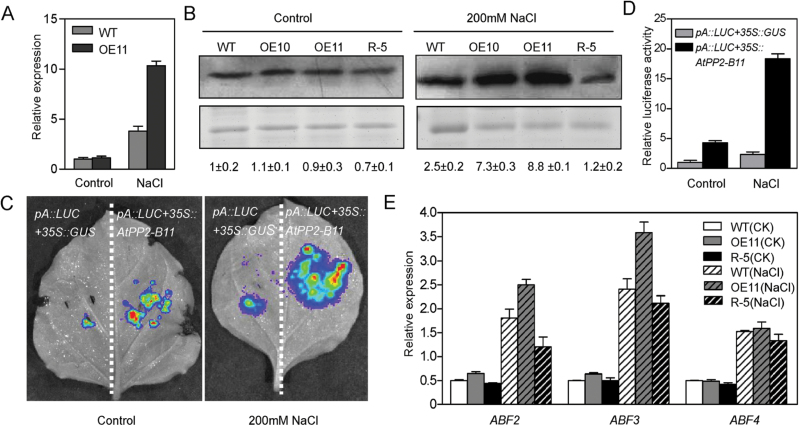
AtPP2-B11 regulates the levels of AnnAt1 under salt stress conditions. (A) Transcript level of *AnnAt1* in the OE11 transgenic and wild-type plants under normal and salt stress conditions. Two-week-old seedlings of the OE11 transgenic and wild-type plants were treated with water or 200mM NaCl for 6h. Total RNA was obtained and analysed by real-time RT-PCR. (B) Protein levels of AnnAt1 in *AtPP2-B11* transgenic and wild-type plants under normal and salt stress conditions. Coomassie Brilliant Blue R250 staining was used to show protein loading levels. (C, D) AnnAt1 promoter activities (C) and relative LUC fluorescence (D) after co-expressing *AtPP2-B11* in the leaves of 4-week-old *N. benthamiana* plants compared with the control. (E) Transcription levels of *ABF2/3/4* in 2-week-old *AtPP2-B11* transgenic and wild-type plants under normal (CK) or salt stress (NaCl) conditions. (This figure is available in colour at *JXB* online.)

Based on the results of iTRAQ analysis, we focused on the highly upregulated and downregulated proteins. Interestingly, among the eight *Arabidopsis* annexins, three members in the OE11 plants were remarkably increased compared with the wild-type plants upon salt stress. As shown in Table 1, AnnAt1, AnnAt2, and AnnAt3 were upregulated with fold changes of 2.576, 2.667, and 3.593, respectively. To confirm the relationship between *AtPP2-B11* and the annexins, the transcript level of *AnnAt1* was detected using real-time RT-PCR analysis, since *AnnAt1* plays a dominant role among eight annexins in *Arabidopsis*. As shown in Fig. 6A, no significant difference in the *AnnAt1* mRNA level was observed between the OE11 and wild-type plants under normal conditions. After 200mM NaCl treatment for 6h, the *AnnAt1* mRNA expression was more induced in OE11 than in wild-type plants (Fig. 6A). Moreover, Western blot analysis showed that AnnAt1 was significantly induced in the *AtPP2-B11* overexpressing lines but depressed in the R-5 line under salt stress conditions (Fig. 6B). To further confirm that *AtPP2-B11* indeed upregulated the expression of *AnnAt1*, we used a LUC reporter assay to examine *AnnAt1* promoter activity after co-expressing *AtPP2-B11* in leaves of 4-week-old *N. benthamiana* plants, whereas co-expressing *pA::LUC* and *35S::GUS* served as a negative control. Consistent with the real-time RT-PCR results (Fig. 6A), *AnnAt1* promoter activity was induced in *N. benthamiana* epidermal cells when co-expressed with *AtPP2-B11* in comparison with the control (Fig. 6C, D). These results suggested that AtPP2-B11 functions in the salt stress response probably by regulating the expression of AnnAt1.

**Table 1.** Differentially expressed annexins in response to salt stress

Gene name	Protein ID	Locus tag	Fold change	Descriptions
*AnnAt1*	IPI00537995	At1g35720	2.576	Response to stimulus; antioxidant activity
*AnnAt2*	IPI00535210	At5g65020	2.667	Response to stimulus
*AnnAt3*	IPI00519595	At2g38760	3.593	Response to stimulus; antioxidant activity

Promoter analysis of *AnnAt1* revealed the presence of numerous *cis*-regulatory elements, including ABRE [PyACGT(tt)GG/TC at position 156], a classical element for the abiotic stress response in plants. *AREB1/ABF2*, *ABF3*, and *AREB2/ABF4*, which are basic leucine zipper-type transcription factors, could bind to the ABRE *cis*-element involved in ABA signalling to regulate the stress response (Fujii *et al.*, 2007; Lee *et al.*, 2010). In order to determine whether *ABF2*, *ABF3*, and *ABF4* affect the expression of *AnnAt1*, the transcription levels of *ABF2*, *ABF3*, and *ABF4* were analysed by real-time RT-PCR in the OE11, R-5, and wild-type plants under both normal and salt stress conditions. As shown in Fig. 6E, the expression of *ABF2* and *ABF3* was induced in OE11 compared with that in the wild-type and R-5 plants when treated with 200mM NaCl for 6h, whereas the expression of *ABF2* and *ABF3* was quite similar under normal conditions. The expression level of *ABF4* was indistinguishable between the wild-type and transgenic plants under the same growth conditions. These results indicated that *ABF2* and *ABF3*, but not *ABF4*, regulated by *AtPP2-B11*, contributed to the high level of *AnnAt1* in the OE11 line under salt stress conditions, leading to the salt stress tolerance of *AtPP2-B11* overexpressing plants.

### *AtPP2-B11* reduces salinity-induced ROS accumulation

**Fig. 7.**
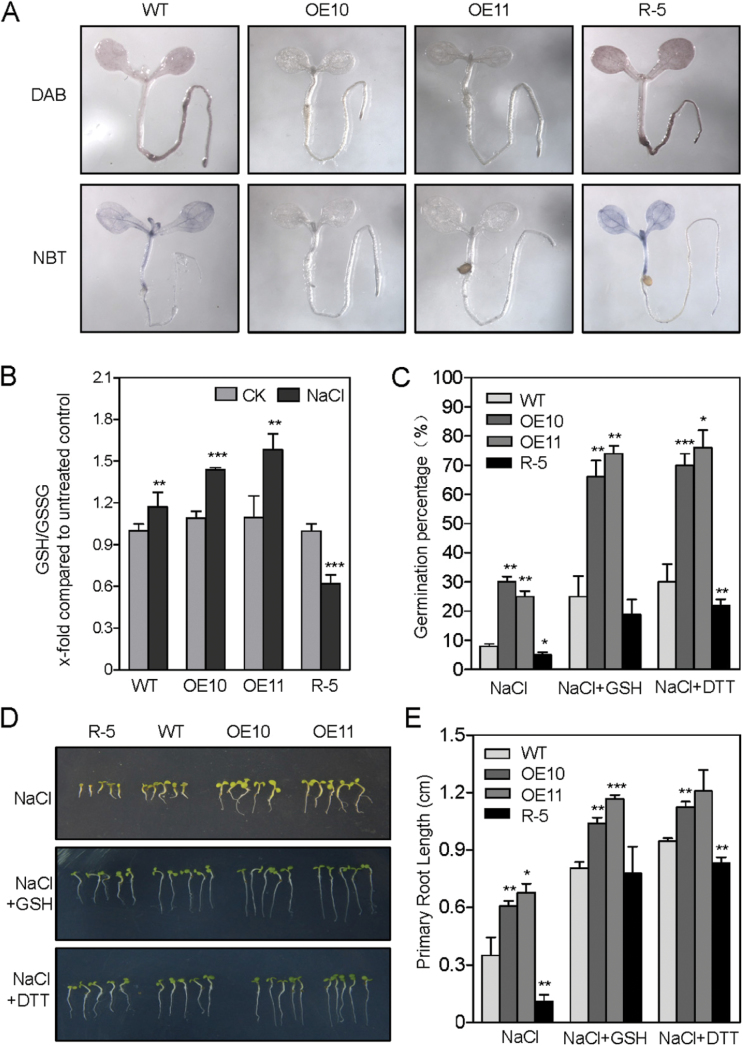
*AtPP2-B11* altered cellular redox homeostasis in *Arabidopsis*. (A) ROS histological staining analysis of *AtPP2-B11* transgenic plants and wild-type plants. The H_2_O_2_ and O^2–^ accumulation in seedlings of OE10, OE11, R-5 and wild-type plants were analysed by DAB and NBT staining, respectively. (B) GSH/GSSG ratio in 2-week-old OE10, OE11, R-5 and wild-type seedlings under normal (CK) or salt stress (NaCl) conditions. (C) Seed germination percentages of *AtPP2-B11* transgenic and wild-type plants grown on ½ MS medium containing 200mM NaCl or 200mM NaCl plus 600 µM GSH or 300 µM DTT 2 d after germination. (D) Salt responses of *AtPP2-B11* transgenic and wild-type plants grown on ½ MS medium containing 200mM NaCl or 200mM NaCl plus 600 µM GSH or 300 µM DTT grown for 2 weeks. (E) Root length of *AtPP2-B11* transgenic and wild-type plants grown on ½ MS medium containing 200mM NaCl or 200mM NaCl plus 600 µM GSH or 300 µM DTT grown for 2 weeks. (This figure is available in colour at *JXB* online.)

iTRAQ analysis revealed that many redox proteins were upregulated in OE11 plants under salt stress (Supplementary Table S3). Considering that *AtPP2-B11* overexpressing lines also enhanced the expressions of annexins, and that annexins act as a molecular link between abiotic stress and ROS signalling, we asked whether the redox homeostasis of *AtPP2-B11* transgenic lines was also altered. In order to verify our hypothesis, ROS accumulation in *AtPP2-B11* transgenic *Arabidopsis* was analysed. The results of DAB staining showed that H_2_O_2_ levels were not significantly different in these seedlings under normal conditions (data not shown). Notably, the OE10 and OE11 seedlings accumulated less H_2_O_2_ compared with the wild-type plants when treated with 200mM NaCl, whereas the accumulation of H_2_O_2_ in the R-5 seedlings was significantly elevated (Fig. 7A). In addition, we also determined O^2–^ levels using NBT staining. The results were similar to those of DAB staining: salinity caused an increase in NBT staining in the R-5 plants, especially in the cotyledons (Fig. 7A). Moreover, the ratio of reduced glutathione (GSH) to oxidized glutathione (GSSG) was measured, and the results showed that OE10 and OE11 had higher ratios of GSH:GSSG compared with wild-type plants in salt stress conditions, which was consistent with the results of DAB and NBT staining analysis (Fig. 7B).

As high levels of ROS in plants can be eliminated by the antioxidants GSH or dithiothreitol (DTT), we further determined whether the salt-sensitive phenotype of the R-5 line could be rescued by GSH or DTT. The sterilized seeds of OE10, OE11, R-5 and wild-type plants were plated on ½ MS growth medium containing 200mM NaCl, 200mM NaCl with 600 µM GSH, or 200mM NaCl with 300 µM DTT. As shown in Fig. 7D, high salinity inhibited post-germination seedling growth on ½ MS medium containing 200mM NaCl. However, when we added 600 µM GSH or 300 µM DTT to ½ MS medium containing 200mM NaCl, the salinity hypersensitivity was rescued during both the germination and post-germination seedling growth stages (Fig. 7C–E). Therefore, we concluded that *AtPP2-B11* might positively regulate the redox genes to reduce ROS levels under salt stress conditions.

### *AtPP2-B11* maintains Na^+^ homeostasis under salt stress conditions

**Fig. 8.**
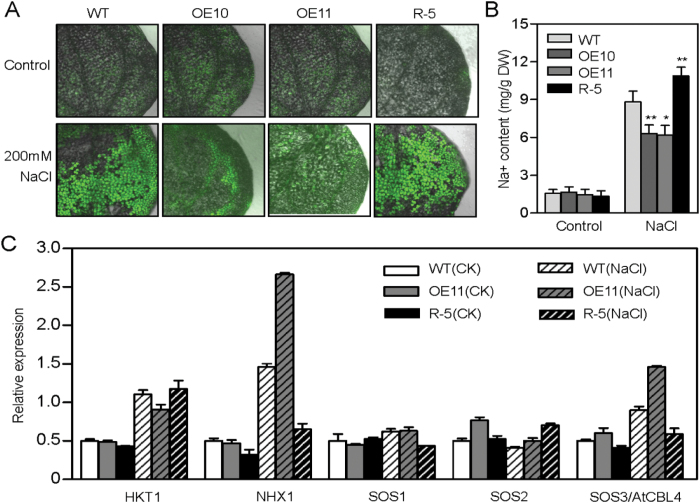
Na^+^ accumulation in *AtPP2-B11* transgenic and wild-type plants exposed to salt stress. (A) Na^+^ accumulation in the cotyledons of 2-week-old *AtPP2-B11* transgenic and wild-type seedlings treated with water as control or with 200mM NaCl for 6h. Na^+^ was visualized using CoroNa Green dye by laser-scanning confocal microscope. (B) Na^+^ content in 2-week-old *AtPP2-B11* transgenic and wild-type seedlings treated with water (control) or 200mM NaCl for 6h. (C) Real-time RT-PCR analysis of genes involved in Na^+^ homeostasis. Total RNA from 2-week-old *AtPP2-B11* transgenic and wild-type seedlings was extracted and analysed by real-time RT-PCR. The graphs indicate the fold induction of *HKT1*, *NHX1*, *SOS1*, *SOS2*, and *SOS3* in response to salt stress compared with the control (CK). Mean values from three independent technical replicates were normalized to the levels of an internal control, *GAPDH*. Error bar indicates SD (*n*=3). (This figure is available in colour at *JXB* online.)

To understand the physiological role of *AtPP2-B11* overexpression in enhancing tolerance to salt stress, the accumulation of Na^+^ was visualized in 2-week-old plants by confocal microscopy using CoroNa Green dye (a green fluorescent indicator of Na^+^). No differences were observed in the Na^+^ fluorescence of the transgenic lines OE10, OE11, and R-5 and the wild-type plants under normal conditions. When treated with 200mM NaCl for 6h, the fluorescence of OE10 and OE11 was barely detectable, while intense fluorescence could clearly be observed in leaves of the wild-type and R-5 plants (Fig. 8A). To confirm the above results, Na^+^ levels were also measured using atomic absorption. OE10 and OE11 contained lower concentrations of Na^+^ than the R-5 line and wild-type plants, which was consistent with the results of Na^+^ fluorescence analysis (Fig. 8B).

As *AtPP2-B11* altered the Na^+^ content under high salinity, we determined whether the expression of genes involved in maintaining Na^+^ homeostasis was also affected. The mRNA levels of *HKT1*, *NHX1*, *SOS1*, *SOS2*, and *SOS3* were determined by real-time RT-PCR analysis. As shown in Fig. 8C, under salt stress conditions, the expression levels of *SOS3* and *NHX1* in OE11 were relatively higher compared with those in the wild-type and R-5 plants, and these proteins are believed to be involved in transporting Na^+^ out of the cells or to vacuoles to relieve Na^+^ toxicity. Thus, *AtPP2-B11* functions in the salt stress response at least partially by indirectly regulating the transcription of genes such as *NHX1* and *SOS3* to maintain Na^+^ homeostasis.

## Discussion

The F-box protein family is large, functionally diverse, and distributed among all eukaryotes, and plays important roles in protein degradation. In particular, several F-box proteins have been identified as being involved in regulating hormone signalling pathways. For example, SCF^TIR1^ participates in the auxin signalling pathway by degrading the transcriptional repressor AUX/IAA family in the presence of auxin (Gray *et al.*, 2001; Dharmasiri *et al.*, 2005a, b). Similar to *TIR1*, the F-box protein *AFB1-3* also functions as a negative regulator in the auxin response (Walsh *et al.*, 2006). In gibberellin signalling, SCF^SLY^ targets DELLA proteins for degradation to alleviate inhibition of gibberellin-regulated growth mediated by DELLAs (McGinnis *et al.*, 2003; Gomi *et al.*, 2004). The *GIBBERELLIN-INSENSITIVE DWARF2* (*GID2*) gene in rice is a putative orthologue of *Arabidopsis* SLY (Gomi *et al.*, 2004). In addition, the F-box genes *COI1* and *EBF1/EBF2* are receptors of jasmonate and ethylene, respectively (Potuschak *et al.*, 2003; Sheard *et al.*, 2010). *COI1* participates in wound- and methyl jasmonate-induced secondary metabolism by controlling the activity of *RPD3b*, a histone deacetylase, and other proteins (Devoto *et al.*, 2002, 2005; Xu *et al.*, 2002). Besides functioning in hormone signalling pathways, F-box proteins also participate in various processes of plant growth and development, such as *EID1* and *AFR* involved in phyA-mediated light signalling, *ZTL* involved in photomorphogenesis, and *UFO* involved in floral development in *Arabidopsis* (Buche *et al.*, 2000; Dieterle *et al.*, 2001; Schultz *et al.*, 2001; Durfee *et al.*, 2003; Harmon and Kay, 2003; Ni *et al.*, 2004). Some S-locus F-box genes also play key roles in controlling the pollen function of self-incompatibility (Wang *et al.*, 2004; Ushijima *et al.*, 2004; Sonneveld *et al.*, 2005). AtTLP9 is an *Arabidopsis* TUBBY-like protein that interacts with ASK1, and alteration of *AtTLP9* modulates the plant’s sensitivity to ABA during seed germination and early seedling development (Lai *et al.*, 2004). The rice F-box gene *OsFbx352* is involved in glucose-delayed seed germination (Song *et al.*, 2012). However, studies of the F-box genes involved in abiotic stresses are limited. In *Arabidopsis*, *EDL3* regulates anthocyanin accumulation under drought stress, and *EDL3* functions as a positive regulator in seed germination, root growth, and flowering in ABA-dependent pathway (Koops *et al.*, 2011). The F-box protein DOR, which interacts with ASK14 and CUL1, functions as a novel inhibitory factor for ABA-induced stomatal closure under drought stress in *Arabidopsis* (Zhang *et al.*, 2008). *Arabidopsis* MAX2 plays a key role in plant responses to abiotic stress, and *max2* mutants display strong hypersensitivity to drought stress at the seedling and adult stages (Bu *et al.*, 2014). To our knowledge, few F-box proteins mediating the salt stress response in plants have been reported. In the present study, we found that F-box protein AtPP2-B11, which functioned as an SCF E3 ligase, played a significant role in the salt stress response. Real-time RT-PCR and Western blot analysis suggested that AtPP2-B11 was strongly induced by salt stress (Fig. 1). The *AtPP2-B11* overexpressing plants exhibited more tolerance to salt stress than the wild-type plants at the germination, seedling, and adult stages, whereas the RNAi line was more sensitive to salt stress (Figs 2–4 and Supplementary Fig. S7-1–S7-4). Therefore, we speculate that a novel regulation pathway may exist by regulating some factors by *AtPP2-B11* under salt stress conditions. In order to confirm this hypothesis, iTRAQ analysis was performed to identify the proteins that were regulated by *AtPP2-B11* in *Arabidopsis* under salt stress. Among the differentially expressed proteins, many proteins involved in salt and oxidative stresses were upregulated (Supplementary Tables S2 and S3), suggesting that AtPP2-B11 may alter the redox state of the plants to resist the environment salt stress. Real-time RT-PCR was also performed to check the transcription levels of oxidative stress response genes upregulated by AtPP2-B11, and the high expression levels of these oxidative stress genes could contribute to the salt stress tolerance in *AtPP2-B11* overexpressing plants. Among the 12 genes selected, the transcription patterns of nine genes were consistent with their protein levels (Supplementary Fig. S3). The agreement between the results of real-time RT-PCR and iTRAQ were ~75%, indicating that some inconsistency between the transcription and translation level occurred. Protein modifications after translation, such as phosphorylation, acetylation, methylation, and ubiquitination, are important regulating pathways in plants, affecting the expression levels of proteins. Secondly, the interaction between proteins also influences the protein expression level. Moreover, after transcription, the mRNA level of genes is also influenced by many factors, such as microRNAs, long non-coding RNAs, small interfering RNAs and long non-coding RNAs.

Among the differentially expressed proteins in iTRAQ analysis, three annexins were significantly induced in *AtPP2-B11* overexpressing plants by salt stress compared with the wild-type plants (Table 1). Given that the *Arabidopsis* annexin protein family has eight members, we propose a possible relationship between *AtPP2-B11* and annexins under salt stress. Because the expression of *AnnAt1* was upregulated by various stresses, and it was dominant among the eight annexins (Clark *et al.*, 2001), we focused on *AnnAt1* for detecting the relationship between *AtPP2-B11* and salt stress. Real-time RT-PCR and Western blot analysis showed that the mRNA and protein levels of AnnAt1 were induced in *AtPP2-B11* overexpressing plants under salt stress compared with those in wild-type plants (Fig. 6A, B). *AnnAt1* promoter activities were also induced in *N. benthamiana* epidermal cells when co-expressed with *AtPP2-B11* compared with the control, consistent with the real-time RT-PCR results (Fig. 6C, D). The increase in *AnnAt1* mRNA levels in *AtPP2-B11* transgenic and wild-type plants was higher than the increase in protein levels, suggesting that the regulation of *AnnAt1* expression between transcription and translation is complex. Alternatively, the discrepancy observed may be due to the use of different experimental methods (real-time RT-PCR for the transcription level and Western blotting for the protein level). It has been reported that plant annexins are capable of forming a Ca^2+^-permeable conductance in an oxidized membrane simulating ROS signalling caused by abiotic stress (Mortimer *et al.*, 2008). In agreement with the induced expression of *AnnAt1*, the *AtPP2-B11* overexpressing lines also exhibited lower ROS levels, indicating that *AtPP2-B11* functions as a signalling mediator between ROS and the salt stress response (Fig. 7 and Supplementary Fig. S7-5). Interestingly, *AtPP2-B11* obviously influenced Na^+^ homeostasis in *Arabidopsis* seedlings under salt stress conditions. Na^+^ fluorescence and Na^+^ content analysis showed that *AtPP2-B11* overexpressing lines accumulated lower Na^+^ than the wild-type and R-5 plants under salt stress conditions (Fig. 8A, B and Supplementary Fig. S7-6). Moreover, the transcript levels of *SOS3* and *NHX1*, which are involved in Na^+^ homeostasis, were higher in OE11 compared with those in the wild-type and R-5 plants, suggesting that high levels of *SOS3* might contribute to transport the excess Na^+^ out of cells, and *NHX1* could transport the remaining Na^+^ to vacuoles in order to avoid Na^+^ toxicity in cells (Fig. 8C). In addition, it was shown that the increased expression of annexins increased the level of Ca^2+^ in cells, and that Ca^2+^ was the secondary message to trigger the stress response (Laohavisit *et al.*, 2013). In *AtPP2-B11* overexpressing plants, a higher level of *AnnAt1* might cause the increased level of Ca^2+^ in cells, and the increased [Ca^2+^]_cyt_ may regulate the Na^+^ homeostasis genes in a feedback manner, such as *SOS3*, a calcium sensor.

It is necessary to identify the substrates of AtPP2-B11 to further understand the cellular functions and regulatory mechanism. E3 ligase degrades the target substrate though the Ub/26S pathway based on direct protein–protein interaction, so the interactions between AtPP2-B11 and the 19 decreased proteins (iTRAQ results) were performed using yeast two-hybrid analysis (Supplementary Table S4, available at *JXB* online). Unfortunately, no protein was identified as interacting with AtPP2-B11 (Supplementary Fig. S4). Our previous study proved that AtPP2-B11 could interact with AtLEA14 by a yeast two-hybrid screening and bimolecular fluorescence complementation assay (Li *et al.*, 2013). However, when treated with salt stress, the Western blot results showed that AtPP2-B11 had no influence on the expression of AtLEA14 at the translation level, suggesting that AtPP2-B11 could not degrade AtLEA14 under salt stress conditions (Supplementary Fig. S5A, available at *JXB* online). On the other hand, AtLEA14 could stabilize the protein level of AtPP2-B11 under normal or salt stress conditions (Jia *et al.*, 2014). A Western blot assay showed that *AtLEA14* overexpressing lines OE4 and OE6 accumulated more AtPP2-B11 protein than the wild-type plants regardless of salt stress treatment (Supplementary Fig. S5B). AtLEA14 was expressed and purified from *E. coli* and then added to the total protein extracted from wild-type plants. The expressed AtLEA14 protein adding to buffer only (no plant extract added) served as a negative control. *In vitro*, AtPP2-B11 became more stable when AtLEA14 was added to the total protein extracted from wild-type plants, and less AtPP2-B11 protein was degraded during the course of incubation (Supplementary Fig. S5C). Moreover, AtPP2-B11 may degrade other unknown transcriptional repressors, which would elevate the expression of salt-responsive genes and result in stress tolerance. Therefore, other unknown AtPP2-B11-interacting partners may be discovered, and they may contribute to this novel salt stress response pathway in plants.

Interestingly, AtPP2-B11 acts as a negative regulator in drought stress (Supplementary Fig. S6, available at *JXB* online) but as a positive regulator in salt stress. In terms of the present data, the different roles of AtLEA14 in drought and salt stresses contributed to the different response of *AtPP2-B11* to these two abiotic stresses. Under drought stress conditions, elevated expression of AtPP2-B11 resulted in the decreased expression of AtLEA14 at the transcriptional and protein levels, and the reduced AtLEA14 could enhance the sensitivity of the *AtPP2-B11* overexpressing plants to drought stress. In addition, *AtPP2-B11* overexpression altered the expression of stress-responsive genes, which led to increased sensitivity to drought stress in seedlings and to water deficit in mature plants. However, under salt stress conditions, AtPP2-B11 did not influence the protein level of AtLEA14, and thus AtPP2-B11 could not degrade AtLEA14 under salt stress conditions, whereas AtLEA14 could stabilize the protein level of AtPP2-B11, and the stabilization of AtPP2-B11 proteins by AtLEA14 reinforced the elimination of negative regulators in salt stress responses. Moreover, AtPP2-B11 responded to drought and salt stresses via different mechanisms in an ABA-independent pathway. We examined the expression of genes in ABA metabolism (*ABA3*, *NCED3*, *CYP707A1*, and *CYP707A2*) and the ABA signalling pathway (*ABI3*/*4*/*5*) under drought and salt stress conditions. The results showed that the expression of these genes had no significant difference in either *AtPP2-B11* transgenic or wild plants. Therefore, it was understandable that *AtPP2-B11* plays opposing roles in drought and salt stresses. This phenomenon indicates the functional diversity and complex regulation mechanism of the same gene in response to different environments in plants.

**Fig. 9.**
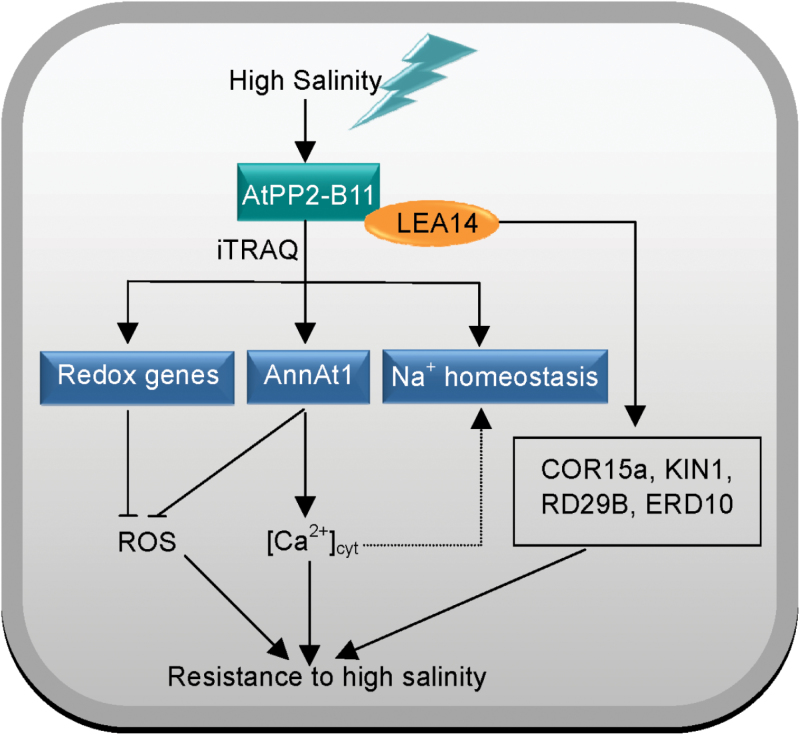
Model of *AtPP2-B11* involvement in the salt stress response. The SCF E3 ligase AtPP2-B11 was remarkably induced at both the transcriptional and translational levels when treated with salt stress. Overexpressing *AtPP2-B11* upregulated the expression of annexins, repressed ROS accumulation, and affected Na^+^ homeostasis under salt stress conditions to elevated salt stress tolerance. In addition, the protein stability of AtPP2-B11 was protected by AtLEA14 under salt stress, and the stable AtPP2-B11 degraded unknown transcriptional repressors, which elevated the expression of salt-responsive genes and subsequent stress tolerance. (This figure is available in colour at *JXB* online.)

Taken together, a model was generated according to our results (Fig. 9). AtPP2-B11, an SCF E3 ligase, was remarkably induced at both the transcriptional and translational levels when treated with salt stress. Overexpressing *AtPP2-B11* upregulated the expression of annexins, repressed ROS accumulation, and affected Na^+^ homeostasis under salt stress conditions to produce elevated salt stress tolerance. In addition, the protein stability of AtPP2-B11 was protected by AtLEA14 under salt stress. However, identification of the substrates of AtPP2-B11 under salt stress remains to be determined, and further experiments should be conducted to explain the detailed regulatory mechanism between *AtPP2-B11* and salt stress.

## Supplementary data

Supplementary data are available at *JXB* online.

Supplementary Fig. S1. AtPP2-B11 mRNA and proteins levels in wild-type and transgenic plants (OE10, OE11, R-5) by real-time RT-PCR (A) and Western blot (B).

Supplementary Fig. S2. Experimental scheme of the iTRAQ analysis.

Supplementary Fig. S3. The verification of 12 selected genes of iTRAQ analysis using real-time RT-PCR.

Supplementary Fig. S4. The interaction between AtPP2-B11 and the 19 decreased proteins (iTRAQ).

Supplementary Fig. S5. The regulation mechanism between AtPP2-B11 and AtLEA14. (A). Protein levels of AtLEA14 in *AtPP2-B11* transgenic and wild-type plants under normal and salt stress conditions. (B). Protein levels of AtPP2-B11 in *AtLEA14* transgenic plants and wild-type under normal and salt stress conditions. (C). AtLEA14 affected the protein stability of AtPP2-B11 *in vitro*.

Supplementary Fig. S6. A model of *AtPP2-B11* involving in drought stress response.

Supplementary Fig. S7. Comparison of *AtPP2-B11* transgenic plants (OE10, OE11, R-5, R-7) and wild-type plants. The germination phenotypes (S7-1), seed germination rates (S7-2), primary root length (S7-3), survival rates (S7-4), ROS histological staining analysis (S7-5), and Na^+^ accumulation (S7-6) of *AtPP2-B11* transgenic plants (OE10, OE11, R-5, R-7) and wild-type plants under normal or salt stress conditions.

Supplementary Table S1. Protein annotation summary.

Supplementary Table S2. Information on the differentially expressed proteins involved in salt stress.

Supplementary Table S3. Information on the differentially expressed proteins involved in oxidative stress.

Supplementary Table S4. Downregulated proteins in iTRAQ analysis.

Supplementary Table S5. The primers used in this study.

Supplementary Data

## Abbreviations:

ABA: abscisic acid
DAB: 3′,3′-diaminobenzidine
DTT: dithiothreitol
GAPDH: glyceraldehyde 3-phosphate dehydrogenase
GO: Gene Ontology
GSH: reduced glutathione
GSSG: oxidized glutathione
iTRAQ: isobaric tag for relative and absolute quantification
MDA: malondialdehyde
NBT: nitro blue tetrazolium
RNAi: RNA interference
ROS: reactive oxygen species
RT-PCR: reverse transcription PCR
SCF: Skp1–Cullin–F-box
SOS: salt overly sensitive
Ub: ubiquitin.
