# Supplementary material for: SCF E3 ligase PP2-B11 plays a positive role in response to salt stress in Arabidopsis
Source: J Exp Bot. 2015 Jun 2;66(15):4683–97. doi: 10.1093/jxb/erv245 (PMC4507775; doi:10.1093/jxb/erv245)
Supplement: Supplementary Data [file supp_66_15_4683__index.html]

SCF E3 ligase PP2-B11 plays a positive role in response to salt stress in Arabidopsis — SCF E3 ligase PP2-B11 plays a positive role in response to salt stress in Arabidopsis — Supplementary Data 

# SCF E3 ligase PP2-B11 plays a positive role in response to salt stress in *Arabidopsis*

## Supplementary Data

Data files

- Supplementary Data - Supplementary Data
- Supplementary Data - Supplementary Data
