# Supplementary material for: SCF E3 ligase PP2-B11 plays a positive role in response to salt stress in Arabidopsis
Source: J Exp Bot. 2015 Jun 2;66(15):4683–97. doi: 10.1093/jxb/erv245 (PMC4507775; doi:10.1093/jxb/erv245)
Supplement: Supplementary Data [file supp_erv245_jexbot149005_file001.pdf]

Figure S1

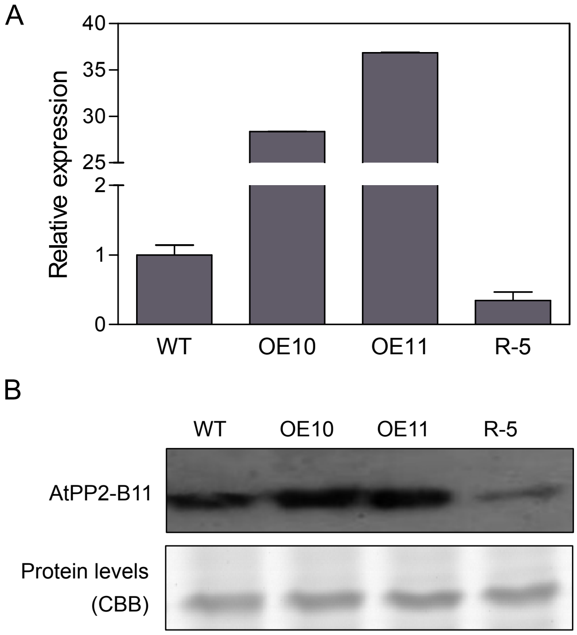
A
40
35
30
25
2
1
0
Relative expression
WT
OE10
OE11
R-5
B
WT
OE10
OE11
R-5
AtPP2-B11
Protein levels
(CBB)

Figure S2

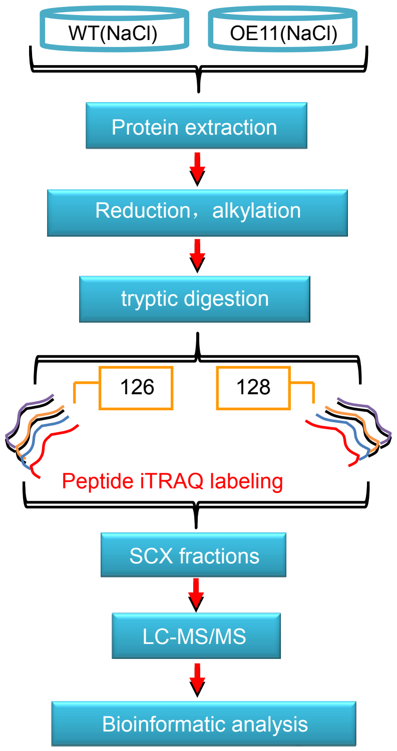

WT(NaCl)
OE11(NaCl)
Protein extraction
Reduction，alkylation
tryptic digestion
126
128
Peptide iTRAQ labeling
SCX fractions
LC-MS/MS
Bioinformatic analysis

Figure S3

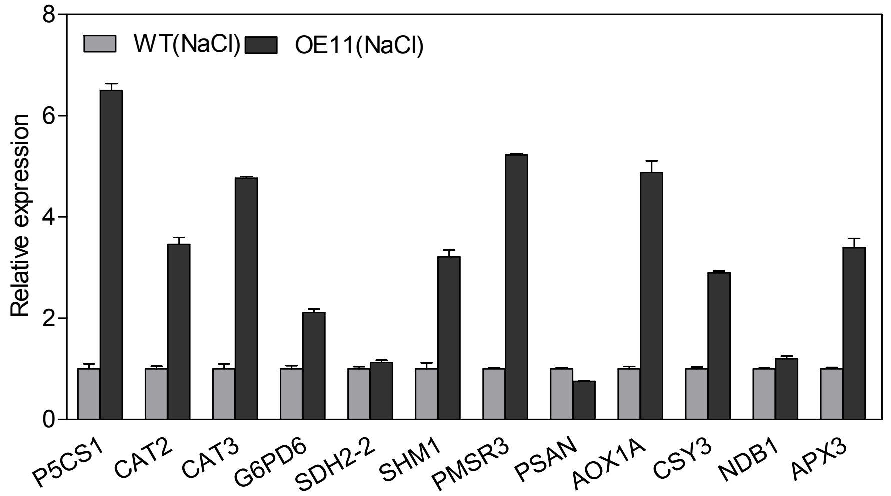
WT(NaCl)
OE11(NaCl)
Relative expression
8
6
4
2
0
P5CS1
CAT2
CAT3
G6PD6
SDH2-2
SHM1
PMSR3
PSAN
AOX1A
CSY3
NDB1
APX3

Figure S4

At2g42210	At1g79260	At2g02170	At5g01650	At3g54640
At5g44500	At1g02410	At2g39770	At1g12210	At2g38730
At4g23860	At1g29840	At4g19540	At1g50320	At5g16990
At2g37640	At1g03000	At5g44340	At5g56090	Control(+)

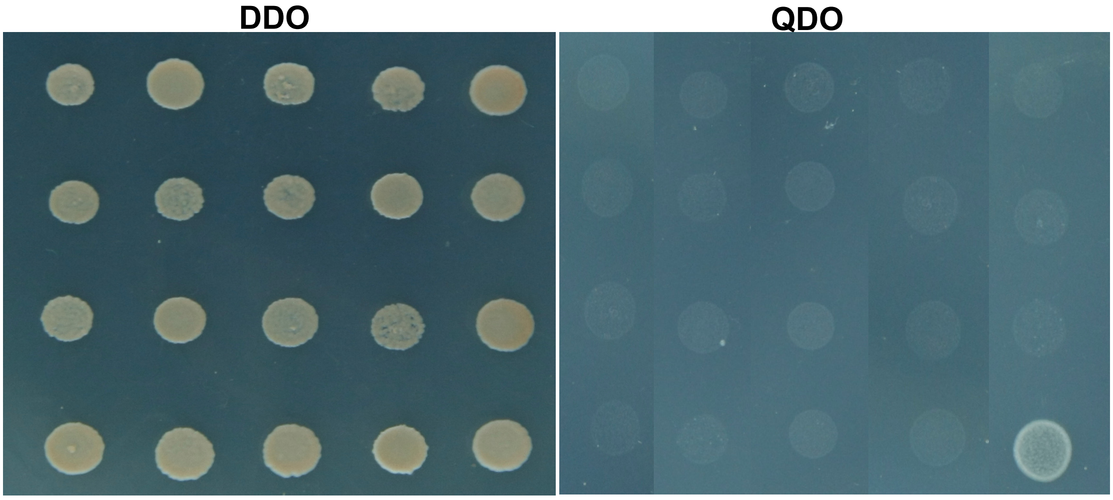

Figure S5

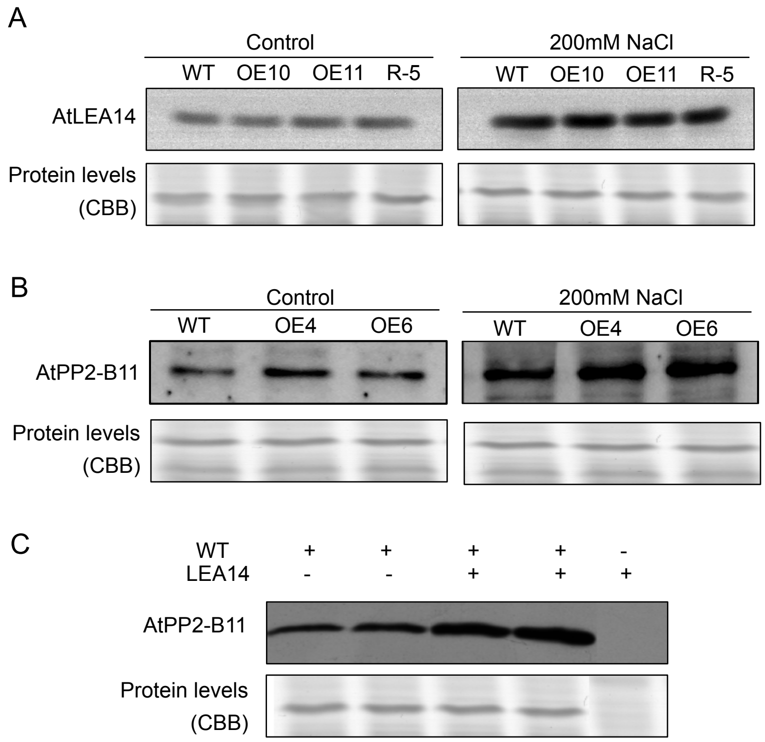
A
Control
WT
OE10
OE11
R-5
200mM NaCl
WT
OE10
OE11
R-5
AtLEA14
Protein levels
(CBB)
B
Control
WT
OE4
OE6
200mM NaCl
WT
OE4
OE6
AtPP2-B11
Protein levels
(CBB)
C
WT + + + + -
LEA14 - - + + +
AtPP2-B11
Protein levels
(CBB)

Figure S6

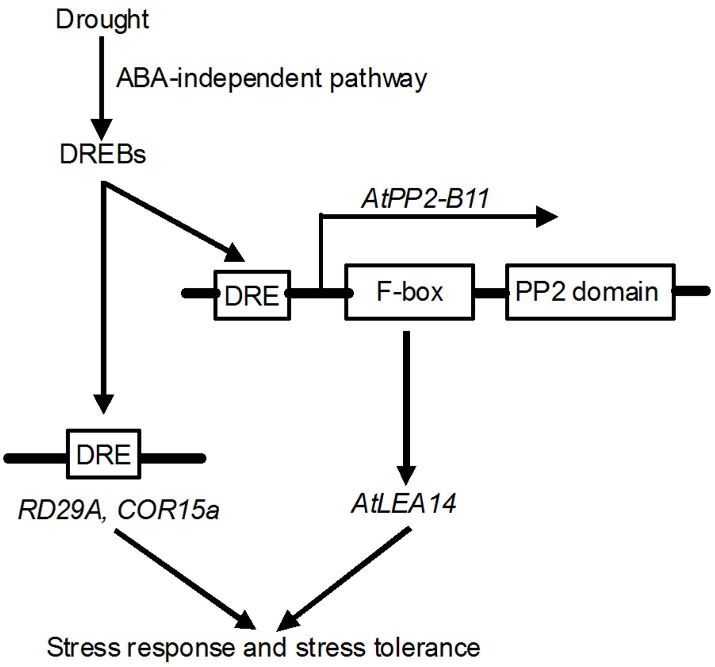
Drought
ABA-independent pathway
DREBs
AtPP2-B11
DRE
F-box
PP2 domain
DRE
RD29A, COR15a
AtLEA14
Stress response and stress tolerance

Figure S7

S7-1

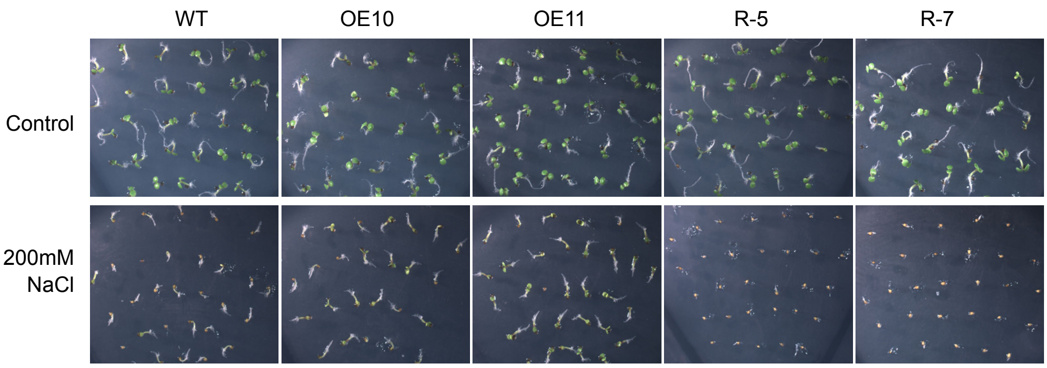

S7-2

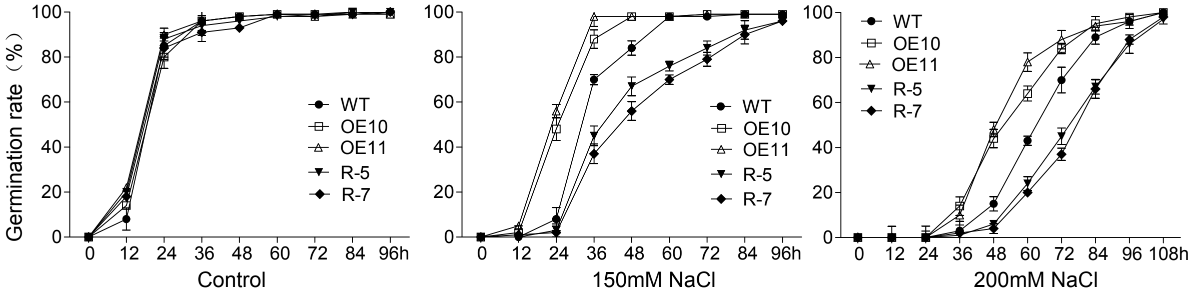

S7-3

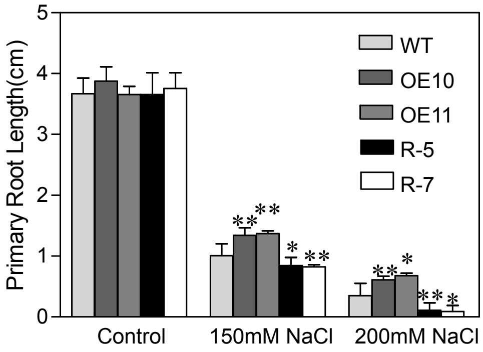

S7-4

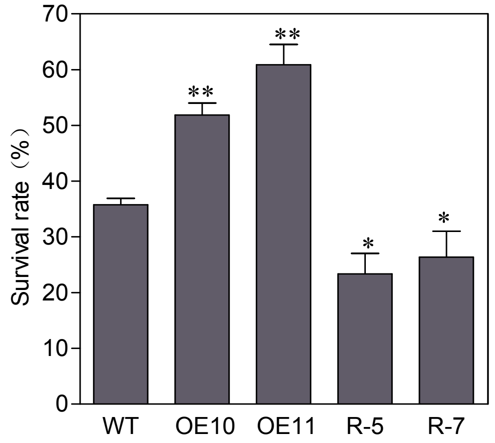

Survival rate（%）
70
60
50
40
30
20
10
0
**
**
*
*
WT
OE10
OE11
R-5
R-7

S7-5

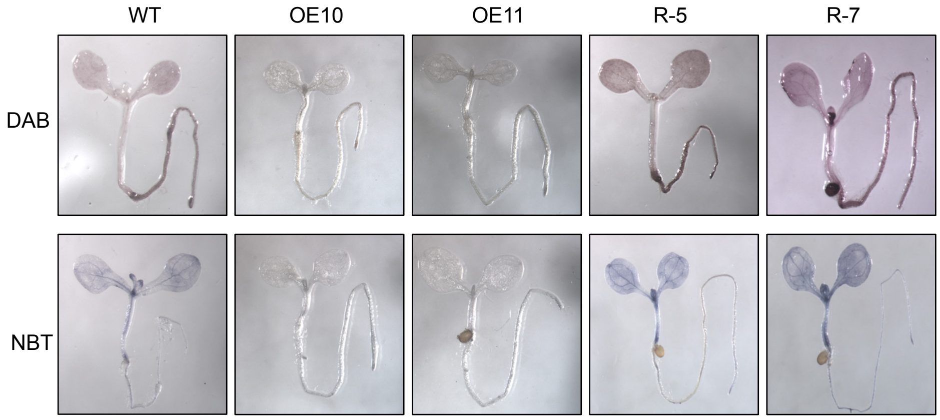

WT
OE10
OE11
R-5
R-7
DAB
NBT

S7-6

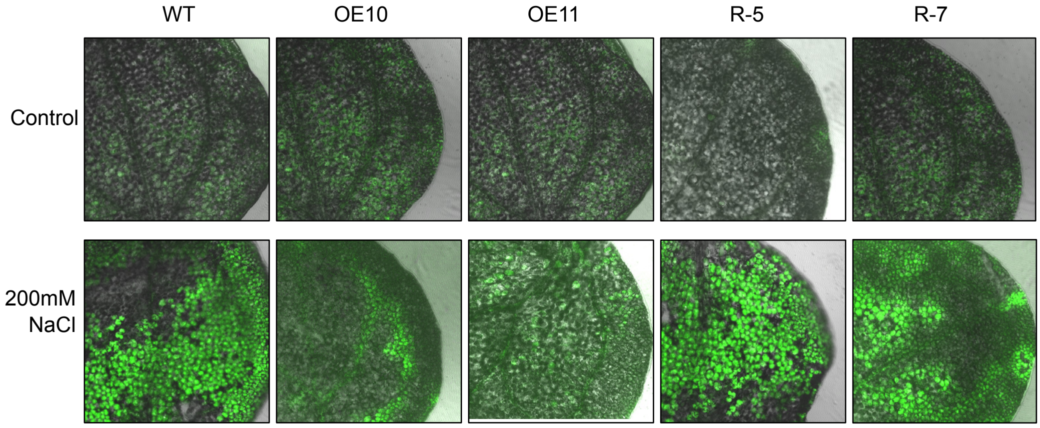

WT
OE10
OE11
R-5
R-7
Control
200mM NaCl
